# Supplementary material for: Retinoic Acid-Containing Liposomes for the Induction of Antigen-Specific Regulatory T Cells as a Treatment for Autoimmune Diseases
Source: Pharmaceutics. 2021 Nov 17;13(11):1949. doi: 10.3390/pharmaceutics13111949 (PMC8620283; doi:10.3390/pharmaceutics13111949)
Supplement: Supplementary file 1 [file pharmaceutics-13-01949-s001.zip › pharmaceutics-1421518-supplementary.pdf]

pharmaceutics

MDPI

Supplementary Materials: Retinoic Acid-Containing Liposomes for the Induction of Antigen-Specific Regulatory T Cells as a Treatment for Autoimmune Diseases

Daniëlle ter Braake, Naomi Benne, Chun Yin Jerry Lau, Enrico Mastrobattista, Femke Broere

Supplements

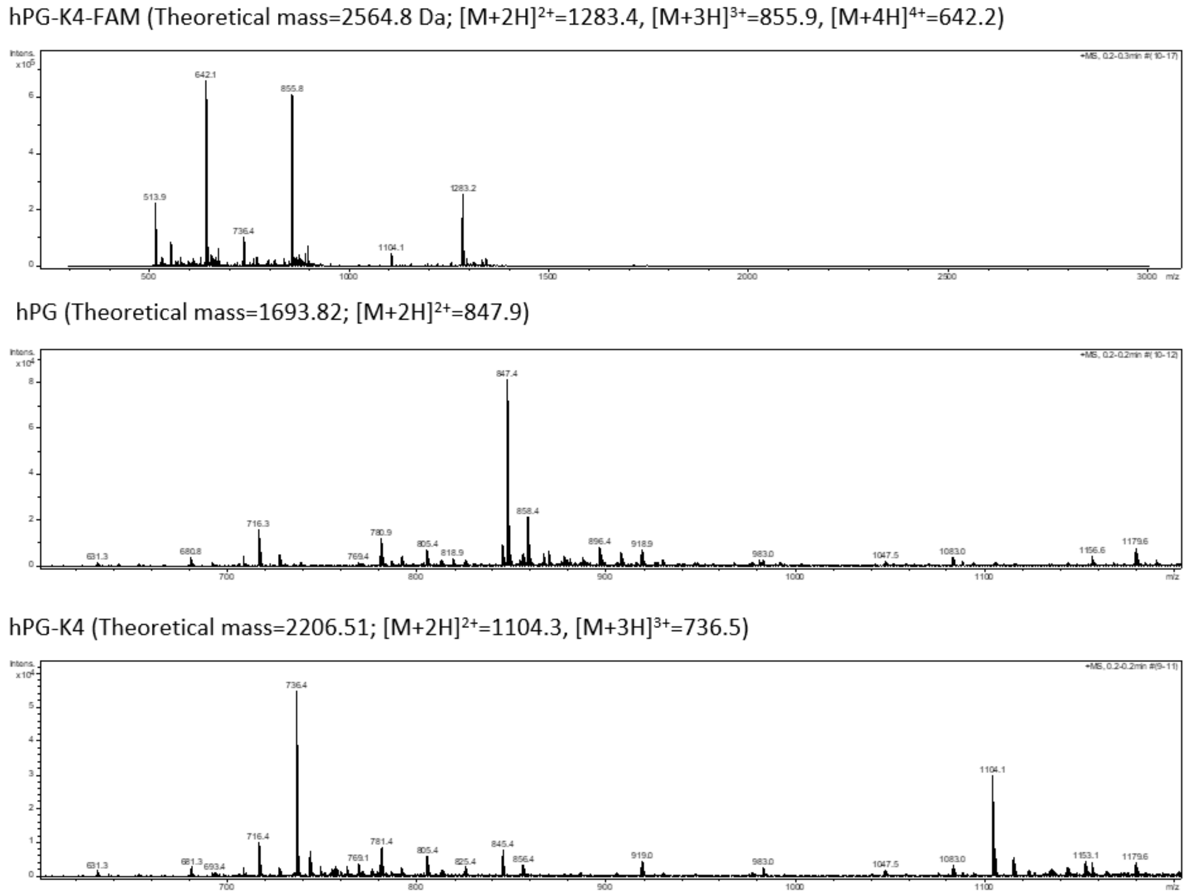

Figure S1. Mass spectrometry analysis of the synthesized peptide products.

Table S1. properties of hPG-FAM liposomes, means.

Formulation	Z-average Diameter (nm)	ζ-potential (mV)	PDI	Encapsulation hPG (%)	Encapsulation RA (%)
hPG-FAM	197.7	−45.6	0.105	34.4	-
hPG-FAM/RA	202.5	−41.3	0.103	38.7	77.3

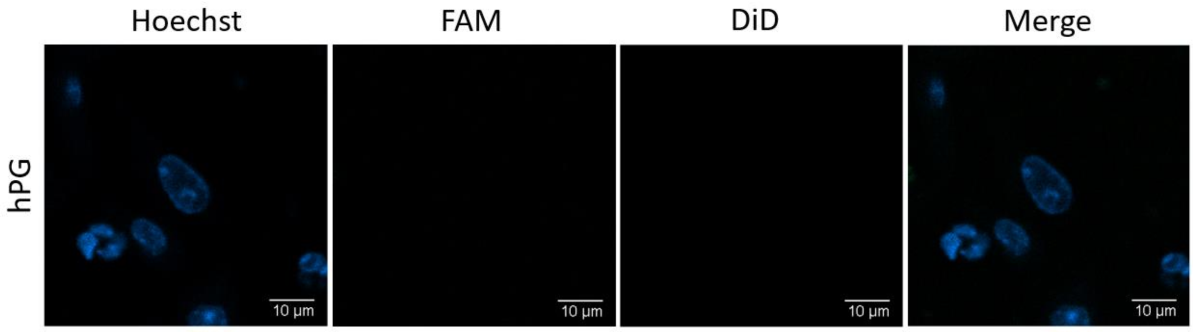

Figure S2. Background fluorescence from unlabeled hPG. BMDCs were cultured from the bone marrow of Balb/c mice. BMDCs were stimulated with LPS and cultured in the presence of 1 µg/mL hPG. After 24 hours incubation, cells were washed carefully to remove unbound liposomes. Briefly before imaging, 5 µg/mL Hoechst dye was added to each well.
